# Urban green roofs host intrinsic resistomes shaped by management but not dominated by pathogenic resistance

**DOI:** 10.1186/s12866-026-05495-6

**Published:** 2026-08-06

**Authors:** Rubén Martínez-Cuesta, Alexandra Craighero, Susanne Walch, Brigitte Helmreich, Michael Schloter, Stefanie Schulz

**Affiliations:** 1 Chair of Environmental Microbiology, TUM School of Life Sciences, Technical University of Munich, Emil-Ramann-Straße 2, Freising, 85354 Germany; 2 Research Unit Comparative Microbiome Analysis, Helmholtz Zentrum Munich, Ingolstädter Landstraße 1, Neuherberg, Oberschleißheim, 85764 Germany; 3 Chair of Terrestrial Ecology, TUM School of Life Sciences, Technical University of Munich, Hans-Carl-von-Carlowitz-Platz 2, Freising, 85354 Germany; 4 Chair of Urban Water Systems Engineering, School of Engineering and Design, Technical University of Munich, Am Coulombwall 3, Garching, 85748 Germany

**Keywords:** Antimicrobial resistance genes, Urban biodiversity, Urban management, Long-read metagenomics, One Health

## Abstract

**Background:**

Urban green roofs are increasingly introduced to enhance urban biodiversity and ecosystem services, yet their role in shaping antimicrobial resistance in cities remains unclear. Using long-read metagenomic sequencing, we characterized antimicrobial resistance genes (ARGs) across an experimental extensive green roof system with plots under four different management regimes specifically designed to test the influence of vegetation and organic amendments, as green waste, which although widely used to improve substrate quality, has been flagged as a potential ARG source.

**Results:**

We detected 62 ARGs across the four management regimes, which were dominated by target-modification and mixed mechanisms conferring resistance to naturally occurring antibiotics such as bacitracin (*bacA*) and rifamycin (*arr*, *rox*, *rph*), rather than efflux-based multidrug resistance, which is typically co-selected by anthropogenic pollutants. The ARGs were mainly chromosomally encoded, with only two ARGs located on plasmids, and associated with non-pathogenic environmental taxa. The management regime had a significant effect on ARG richness, ARG composition and plasmid abundance, but not on average genome size-normalized ARG abundance. We also detected *aph3-II* and *tlmA* as enriched in the unamended samples, which were carried by oligotrophic bacteria, pointing towards microbial competition in a nutrient-limited environment.

**Conclusions:**

Overall, our findings indicate that green roof management supports a substrate resistome driven by ecological constraints rather than clinical threats. However, further research is required to evaluate potential risks and support the safe integration of green roofs within a One Health framework.

**Graphical abstract:**

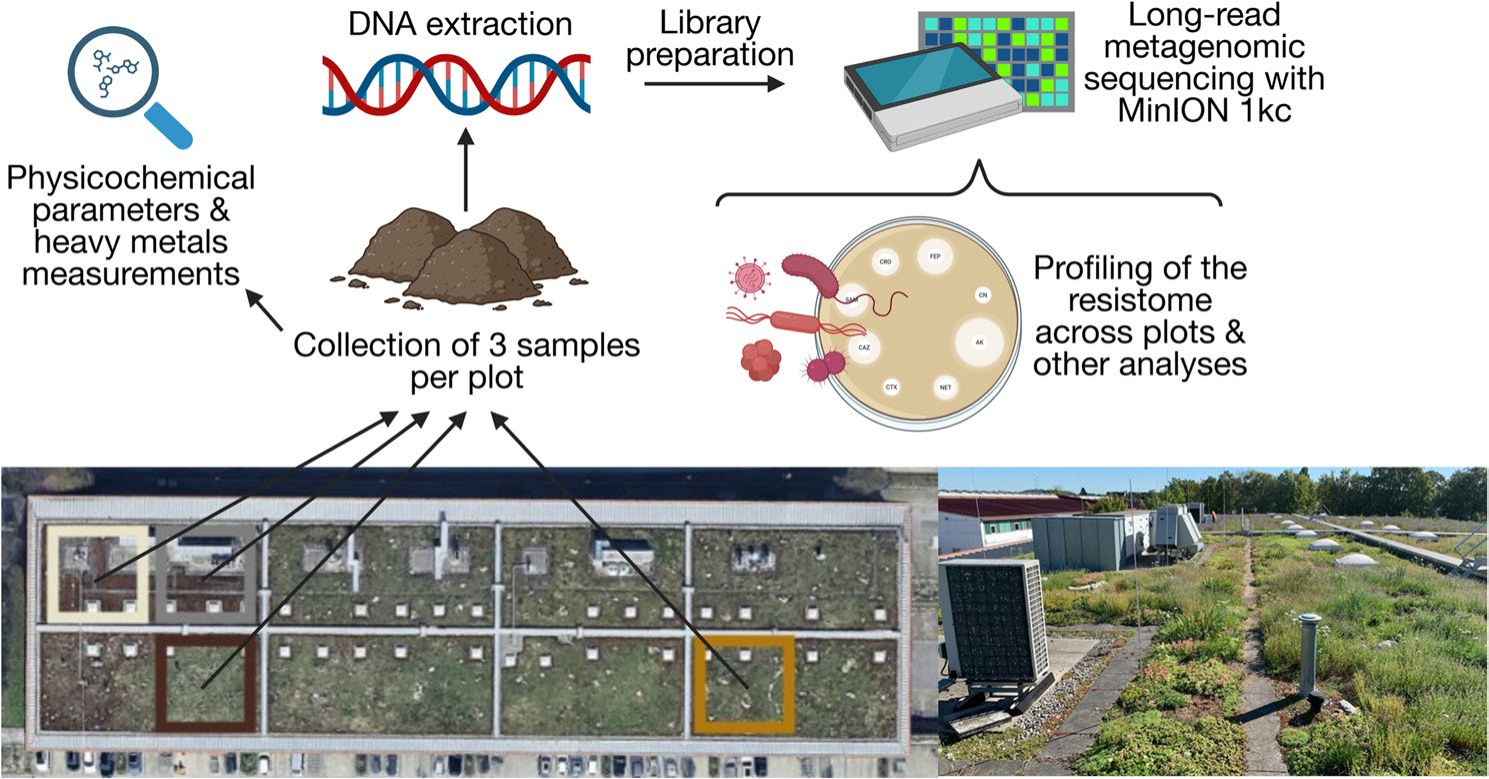

**Supplementary Information:**

The online version contains supplementary material available at 10.1186/s12866-026-05495-6.

## Background

Antimicrobial resistance (AMR) is widely recognized as one of the most pressing global health challenges, with environmental reservoirs increasingly acknowledged as key contributors to resistance emergence and dissemination [1, 2]. Soils host a vast diversity of antimicrobial resistance genes (ARGs), many of which represent intrinsic traits that evolved long before the anthropogenic use of antibiotics [3]. These environmental ARGs can nonetheless pose risks to human and animal health when mobilized and transferred to pathogenic bacteria [4].

Urban environments are of particular concern due to their high levels of disturbance, pollution, and selective pressures, including airborne particulates, heavy metals, and other chemical contaminants that can co-select for antibiotic resistance [5, 6]. At the same time, cities often exhibit reduced microbial diversity, which may favour the presence of pathogens, resistant taxa and horizontal gene transfer [2, 7, 8]. As urbanization increases globally, understanding how urban ecosystem design influences microbial resistance has become a priority for the One Health agenda.

Green roofs are increasingly introduced as nature-based solutions to boost biodiversity, reduce stormwater runoff, regulate microclimates, and strengthen ecosystem functioning [9, 10]. By creating novel soil habitats above ground level, green roofs introduce complex microbial communities into the urban environment. However, these systems are also characterized by harsh and fluctuating conditions, including nutrient limitation, desiccation, and intense microbial competition, which are known to promote antimicrobial resistance [7, 11]. Moreover, as elevated structures in urban settings, green roofs can act as sinks of pollutants, like heavy metals and particles from high-traffic proximity, which can trigger co-selection processes, increasing AMR in the absence of direct antibiotic pressure [12]. This occurs through increased mobile genetic elements (MGE) abundance (co-resistance) or by driving efflux-based multidrug resistance via co-selection with heavy metals [13]. Despite the mentioned risks and their growing prevalence, green roofs remain largely unexplored regarding their potential as reservoirs of ARGs.

Management practices applied to green roofs may further influence resistome development. Organic amendments such as urban green waste compost are commonly used to improve substrate quality, enhance microbial biomass, and support vegetation establishment [9]. However, green waste has also been identified as a source of ARGs in composting systems, raising concerns about its use in urban green infrastructure [14]. Studies in agricultural and urban soils demonstrate that soil properties such as pH, carbon availability, phosphorus and nitrogen inputs strongly shape ARG abundance and composition [15, 16], yet it remains unclear how these drivers operate in engineered ecosystems such as green roofs.

To close this research gap, we used long-read metagenomics to investigate how long-term green roof management strategies influence ARG diversity, abundance, composition, and taxonomic carriers in an experimental green roof system in Munich, Germany. By comparing green waste addition, seeding, and the presence of bulbs or shrubs, we aimed to assess whether biodiversity-enhancing management practices promote ARG proliferation or, instead, they uphold resistomes dominated by intrinsic resistance. Our findings provide a clearer picture of how nature-based solutions influence urban antimicrobial resistance.

## Methods

### Study site and sample collection

The study was conducted on a green roof in Munich, Germany (approx. 48°12′12″N, 11°35′14″E; Fig. S1) managed by *Die Naturgartenplaner* (https://naturgartenplaner.de), which is located 25 m away from a heavy-traffic avenue. The experimental setup was distributed across three distinct roof areas: treatments G1 and G2 were located within a 225 m^2^ area, G3 occupied a separate 225 m^2^ area, and G4 was established within a 278 m^2^ area. All treatments were built upon an initial 8 cm-thick mineral roof substrate. Plots for G1–G3 were constructed in 2018, while G4 was established in 2020. Management varied by treatment: G3 and G4 received a 2 cm layer of green waste compost (equivalent to 4.5 m^3^) with G4 further receiving an additional 7 cm intensive substrate layer. While G1 served as a control with spontaneous vegetation, plots G2–G4 were sown with specific plant species mixtures (Supplementary methods). Further details on the experimental design can be found in Table 1. Three subplots of 1 m^2^ were established in each of the four areas, from which topsoil samples (0–5 cm) were collected and homogenized with a 5 mm sieve and then stored at − 80 °C. Information about physicochemical parameters and heavy metal content of the samples can be found in Table S1.

**Table 1:** Structure, management details and plant species of the green roof plots

Sample	Treatment	Structure	Management	Plant species
G1.1	G1	8 cm extensive substrate	Non	0
G1.2				
G1.3				
G2.1	G2	8 cm extensive substrate	seeding + 5 bulb species	31
G2.2				
G2.3				
G3.1	G3	8 cm extensive substrate + 2 cm green waste compost layer (4.5 m^3^)	seeding + 12 bulb species + 4.5 m^3^ green waste	31
G3.2				
G3.3				
G4.1	G4	8 cm extensive substrate + 7 cm intensive substrate + 2 cm green waste compost layer (4.5 m^3^)	seeding + 7 shrubs + green waste	24
G4.2				
G4.3				

### DNA extraction, library preparation and sequencing

Total DNA was extracted from 0.5 g of soil using the NucleoSpin^®^ Soil Kit (Macherey-Nagel, Düren, Germany) following the manufacturer’s guidelines. The bead beating step was performed with a Precellys24 homogenizer (Bertin Technologies, Montigny-le-Bretonneux, France) and was reduced to 30 s at 5,000 rpm to maintain DNA integrity. Fragments size was checked using a Fragment Analyzer System 5300 (Agilent Technologies, Santa Clara, CA, USA). The Native Barcoding Kit 96 V14 (Oxford Nanopore Technologies Ltd., Oxford, UK) was deployed for the library preparation along with 400 ng from each sample per barcode (in 11 µL). Sequencing was performed within a MinION Mk1C device and the R10.4.1 flow cells (FLO-MIN106) using a final DNA concentration of 6.28 ng/µL. A negative extraction control consisting of the kit reagents was included and sequenced along with the samples.

### Bioinformatic processing

Basecalling and the removal of sequencing adapters were carried out with dorado v0.7.2 (Oxford Nanopore Technologies Ltd., Oxford, UK) using the ‘basecaller’ function with the dna_r10.4.1_e8.2_400bps_hac@v5.0.0 model. Reads were subsequently demultiplexed based on barcodes. Quality filtering was performed using chopper [17] v0.8.0, with a minimum average read quality threshold of 10 and a minimum length of 500 bp. Contaminants were removed by mapping the filtered reads to the latest version of the NCBI human genome reference sequence (GRCh38/hg38; GCF_000001405.40) and the reads in the negative control using minimap2 [18] v2.28 with the ‘map-ont’ parameter.

The long-read-based Argo [19] v0.2.1 profiler, with its curated SARG+ database, was deployed to identify ARGs within the metagenomes and to determine their genomic location (chromosomal or plasmid-borne). These ARGs confer resistance to clinically relevant antibiotics, based on the World Health Organization list [20], as well as to biocides, multidrug efflux systems, and genes associated with veterinary, agricultural and other non-clinical antibiotic use. Following the extraction of ARG-containing reads, taxonomic classification was performed using Centrifuger [21] v1.0.11 with a pre-built index based on the GTDB v226 database. ARG abundances were normalized based on the coverage-based copy number provided by Argo ($\:scov\:=\:\frac{send-sstart}{slen}$) using the following formula: 

   

$$\begin{aligned}&\text{Coverage per Giga-base}\;(\mathrm{CPGB})=\\&\frac{scov}{Total\:bases\:per\:sample}\:x\:1\:\times\:\:{10}^{9}\end{aligned}$$

ARG abundance was further normalized to copies per cell by using SingleM [22] v0.21.0 to estimate the average genome size for each sample.

Filtered reads were assembled in sequential mode using metaFlye [23] v2.9.5, and the resulting contigs were analysed with geNomad [24] v1.8.1 in default settings to detect mobile genetic elements.

### Statistical analysis

All statistical analyses were carried out within R v4.3.2. Differences in average genome size-normalized ARG abundance, ARG richness (unique ARGs detected per sample), bacterial biomass (as C_mic_) and bacterial richness (observed species) across treatments were assessed with one-way ANOVA tests with the “anova_test” function of the rstatix v0.7.2 package, with prior residuals testing with the Levene’s test using the “leveneTest” function of the car v3.1-3 package and the Shapiro-Wilk test. Pairwise differences in ARGs richness between treatments were further assessed with Tukey’s HSD test using the “tukey_hsd” function of rstatix. Results were visualized with ggplot2 v3.5.1.

A hierarchical clustering of the samples based on ARG composition and the associated heatmap of the square-root CPGB were plotted using the pheatmap v1.0.12 package.

Dissimilarity in ARGs composition across samples was estimated using the Bray-Curtis distance within the vegan v2.6-4 package with the “vegdist” function. Ordination was conducted with principal coordinates analysis (PCoA) using the “cmdscale” function of the stats v4.3.1 package. The influence of experimental variables on ARG composition was assessed with PERMANOVA (999 permutations) using the “adonis2” function of the vegan package.

To detect enriched ARGs, we carried out differential abundance analyses using MaAsLin2 [25]. We sequentially set each group as reference across runs. The resulting *p*-values were corrected using the Benjamini-Hochberg method. Finally, the *q*-values and effect sizes of all significantly enriched ARGs were visualized in a volcano plot.

To examine relationships between ARGs abundance and soil physicochemical and nutrient parameters, we computed pairwise Spearman correlation coefficients using the linkET v0.0.7.4 package. Correlation coefficients were visualized with the “qcorrplot” function, applying a red–blue colour scale to highlight positive and negative associations.

To visualize the distribution of ARGs across genera, we focused on the 25 most abundant genera detected in the dataset. All other genera were grouped into the “Other” category. Stacked bar plots were generated using ggplot2.

## Results

Long-read metagenomic sequencing was deployed to characterize antimicrobial resistance genes (ARGs) across green roof soils under four long-term management regimes. These included extensive substrate without seeding or green waste (G1), extensive substrate with seeding (G2), extensive substrate with seeding and green waste addition (G3), and intensive substrate with seeding and green waste addition (G4). A total of 12 soil metagenomes were analysed, comprising 7,426,117 reads after quality filtering (Table S2).

Across all samples, 62 ARGs (42 clinically relevant, 4 used in agriculture, veterinary and other non-clinical settings, and 16 corresponding to biocide, multidrug efflux and others; Table S3) belonging to 17 antimicrobial resistance classes were detected using the Argo profiler at the read level. The most abundant ARG classes were associated with resistance to bacitracin (*bacA*), rifamycins (*arr*, *helR*, *rox*, *rph*), aminoglycosides (a*ph(3’)-II*), biocides (*sugE*), bleomycin (*tlmA*), and defensins (*lysX*). Only two ARGs, providing resistance against beta-lactams (*bla*) and trimethoprim (*dfrB*), were located on plasmids, while the remaining ARGs were chromosomally encoded. Regarding multidrug resistance, 10 ARGs belonging to this class were detected. Of those, *mdtA*, *mmp* and *tap* were the most abundant.

**Fig. 1:**
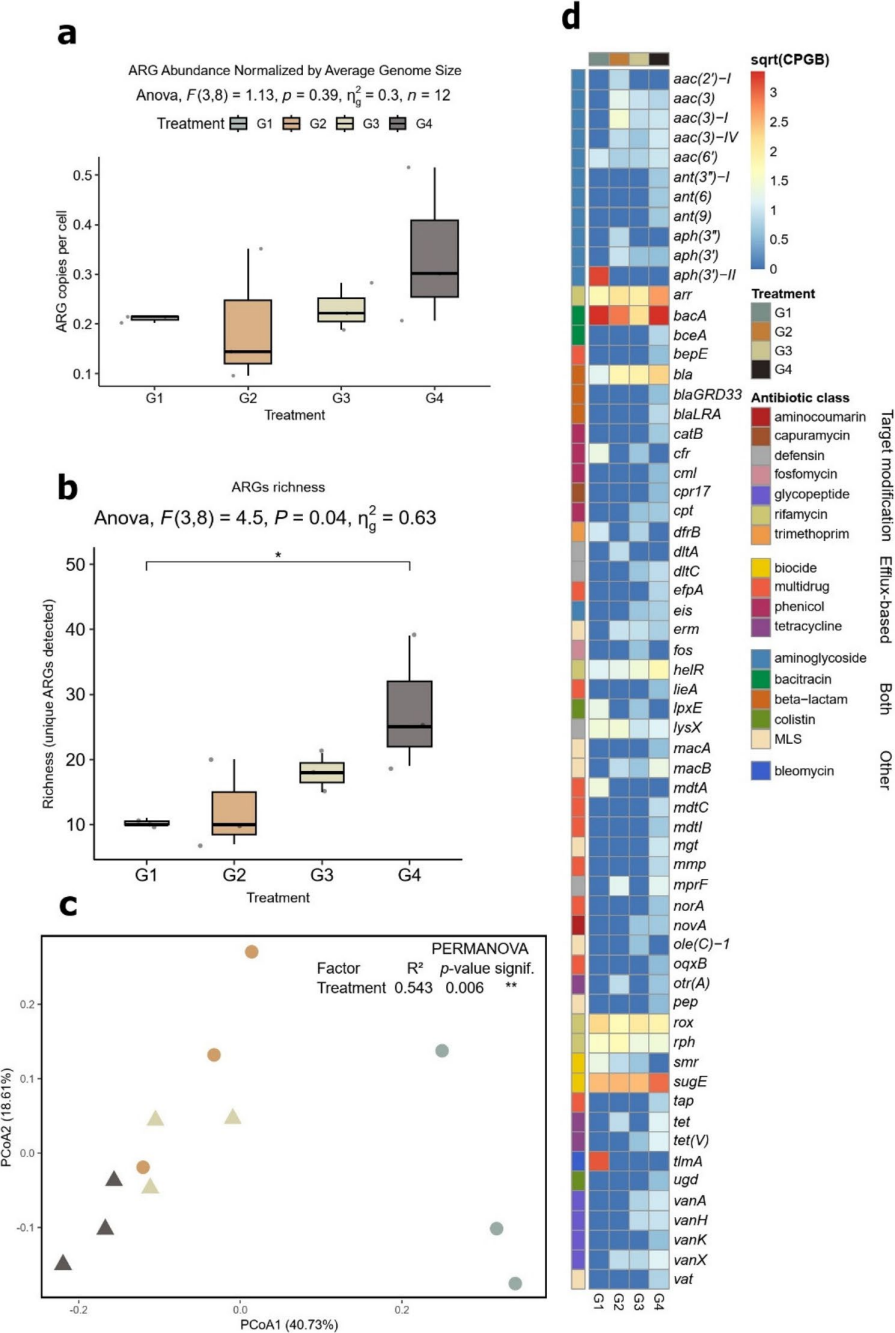
Abundance, richness and composition of ARGsacross the different green roof plots. **a** ARG abundance normalized to average genome size and (**b**) richness of antimicrobial resistance genes (ARGs) across treatments. Abundance was calculated as the sum of coverage per Giga base (CPGB) for all detected ARGs. Richness indicates the number of unique ARGs identified per sample. Statistical significance was determined using ANOVA with Tukey’s HSD for pairwise comparisons. **c** ARG community composition (Bray–Curtis distance). Effects of study variables on ARG composition were assessed with PERMANOVA. Colours indicate treatments, shapes indicate green waste addition. **d** Heatmap of ARG composition (average square-root transformed CPGB values per treatment), with rows annotated by treatment and columns by ARG class (indicating their mechanism of action)

Management treatments had a significant effect on ARG richness (ANOVA; *P* = 0.04; η^2^ = 0.63) Fig. 1a). Plots receiving green waste (G3 and G4) exhibited higher ARG richness compared to non-amended treatments, although only the comparison between G1 and G4 showed a significant *P*-value (Tukey’s HSD; 95% CI: [0.74, 33.92]; *P* = 0.04). Conversely, management treatments did not have a significant effect on ARG abundance, as differences in ARG copies per cell between treatments were not significant (ANOVA; *P* = 0.39; η^2^ = 0.3). Green waste addition also coincided with increased bacterial biomass and higher microbial diversity (Table S1; Fig. S2). Concentrations of the three measured heavy metals (copper, zinc, and lead) remained below the permissible Food and Agriculture Organization of the United Nations (FAO) [26], European Comission (EC) [27] and the German Federal Soil Protection and Contaminated Sites Ordinance (BBodSchV) recommended ranges for soils (Table S4).

ARG composition differed significantly among treatments (Fig. 1c-d). Bray–Curtis dissimilarity analysis revealed distinct clustering by management regime (Fig. 1c), supported by PERMANOVA results indicating a significant treatment effect (*P* = 0.006; R^2^ = 0.543). The robustness of the PERMANOVA results was verified with a PERMDISP analysis (Fig. S3), which yielded no significant differences between treatments (ANOVA; *P* = 0.43; η^2^ = 0.28). Bacitracin (*bacA*) and rifamycin (*arr*, *rox*, *rph*) resistance genes dominated across all treatments, which provide resistance via cell wall synthesis and target site modification in the β-subunit of the RNA polymerase, respectively.

**Fig. 2:**
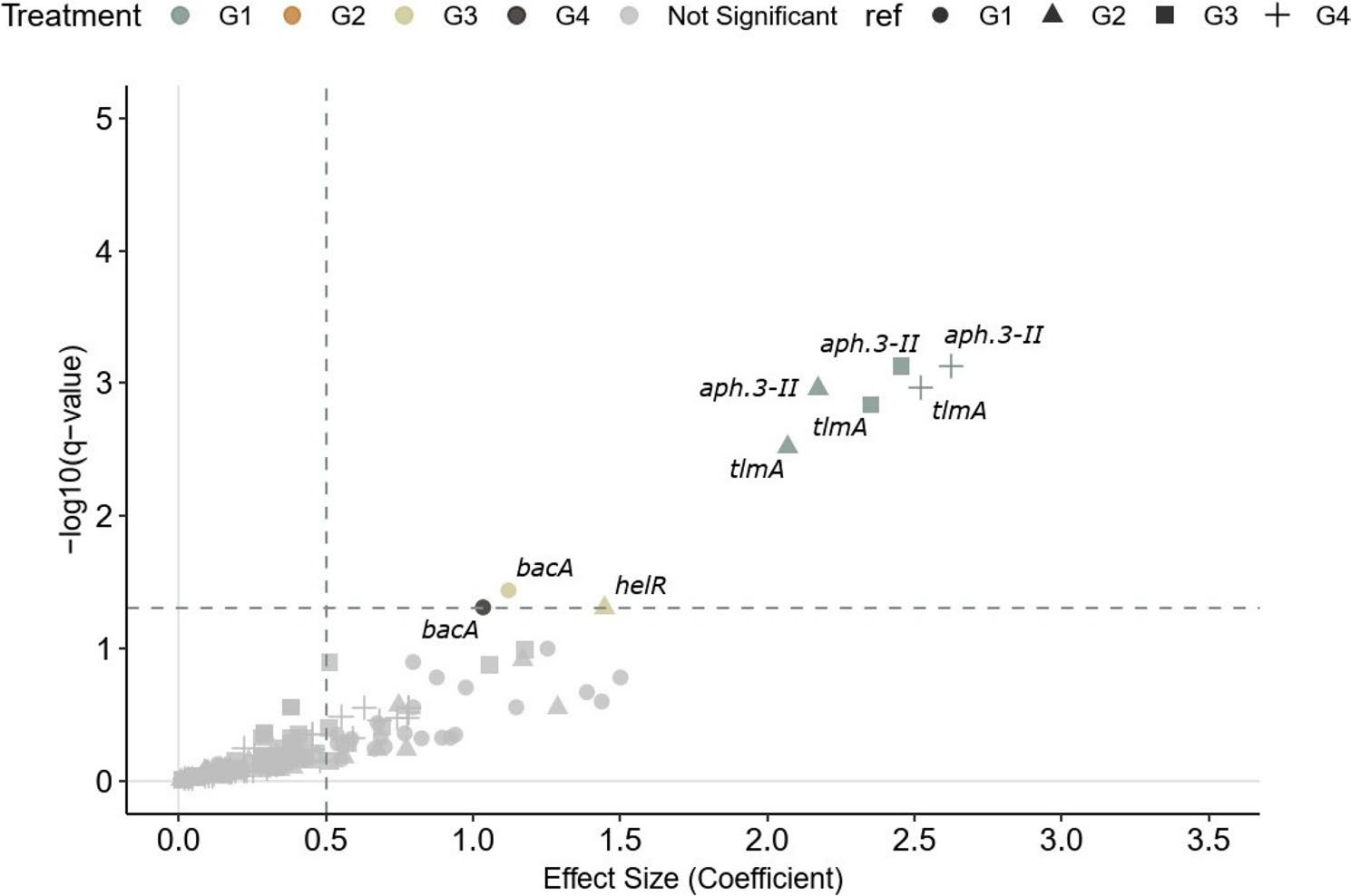
Volcano plot displaying significantly enriched antibiotic resistance genes (ARGs) when combining the results of the MAsLin2 analysis for each group as reference. Effect sizes are represented as Coefficient and significance as −log10 *q*-value. Colours according to group in which the ARG is enriched and shape according to reference group. Non-significant features are represented in light grey. Dashed lines mark thresholds for significance (*q* = 0.05) and effect size (± 1)

**Fig. 3:**
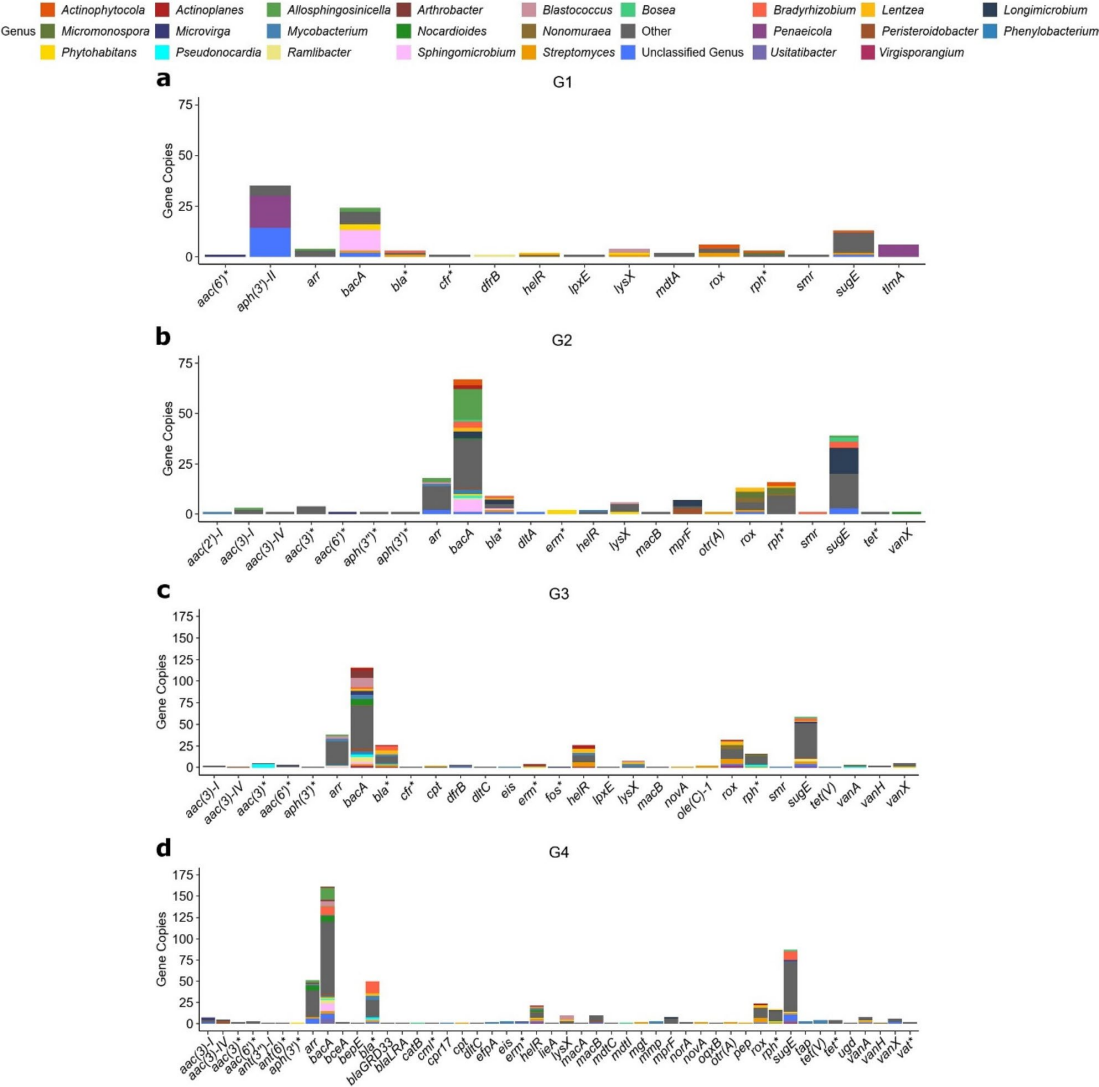
Taxonomic classification of bacterial hosts harbouring the detected antibiotic resistance genes. Gene copies of antibiotic resistance genes (ARGs) detected in green roof metagenomes across treatments (**a**) G1 (**b**) G2 (**c**) G3 and (**d**) G4. Bars are coloured by bacterial genus according to the taxonomic assignation performed with Centrifuger. Only the 25 most abundant genera based on ARG counts across treatments are shown individually. All remaining genera were grouped as “Other”

By performing differential abundance analyses using each group as a reference sequentially, we found distinct enrichment patterns across groups (Fig. 2). In G3 samples, *bacA* and *helR* were significantly enriched compared to G1 and G2, respectively, while *bacA* was also enriched in G4 compared to G1. Conversely, both *aph3-II* and *tlmA* were enriched in G1 samples relative to G2, G3, and G4. We further inspected which taxa carried these enriched ARGs (Fig. 3a-d). In G3 samples, *bacA* was mainly carried by *Blastococcus*, *Arthrobacter*, *Nocardioides*, *Ramlibacter*, *Mycobacterium* and *Microvirga*, whereas in G4 samples it was primarily carried by *Allosphingosinicella*, *Bradyrhizobium*, *Sphingomicrobium*, *Nocardioides* and *Usitatibacter* The *helR* gene enriched in G3 was predominantly associated with *Lentzea*, *Streptomyces*, *Actinoplanes*, and *Mycobacterium*. Finally, in G1, *aph3-II* was mainly associated with *Penaeicola*, *Pseudoalteromonas*, and *Erwinia*, whereas *tlmA* was mainly associated with *Penaeicola Nocardioides* and *Usitatibacter*. Meanwhile, *helR* in G3 was predominantly carried by *Lentzea*, *Streptomyces*, *Actinoplanes* and *Mycobacterium*. Finally, in G1 samples, *aph3-II* and *tlmA* were mainly carried by *Penaeicola*, *Pseudoalteromonas* and *Erwinia* for the former and *Penaeicola* for the latter.

Overall, the taxonomic assignment of ARG-carrying reads revealed that ARGs were predominantly associated with non-pathogenic, soil- and compost-associated bacterial genera. In G1 samples (Fig. 3a), ARGs were mainly carried by *Penaeicola* and *Sphingomicrobium*. G2 samples were dominated by *Longimicrobium* and *Allosphingosinicella* (Fig. 3b), while G3 samples showed a more even distribution across taxa, including *Blastococcus*, *Nocardioides*, *Ramlibacter*, and *Lentzea* (Fig. 3c). In G4 samples, ARGs were primarily associated with *Allosphingosinicella*, *Sphingomicrobium*, *Nocardioides*, and *Bradyrhizobium* (Fig. 3d). No ARGs were detected in members of the ESKAPE pathogen group, such as *Klebsiella pneumoniae* or *Pseudomonas aeruginosa*.

**Fig. 4:**
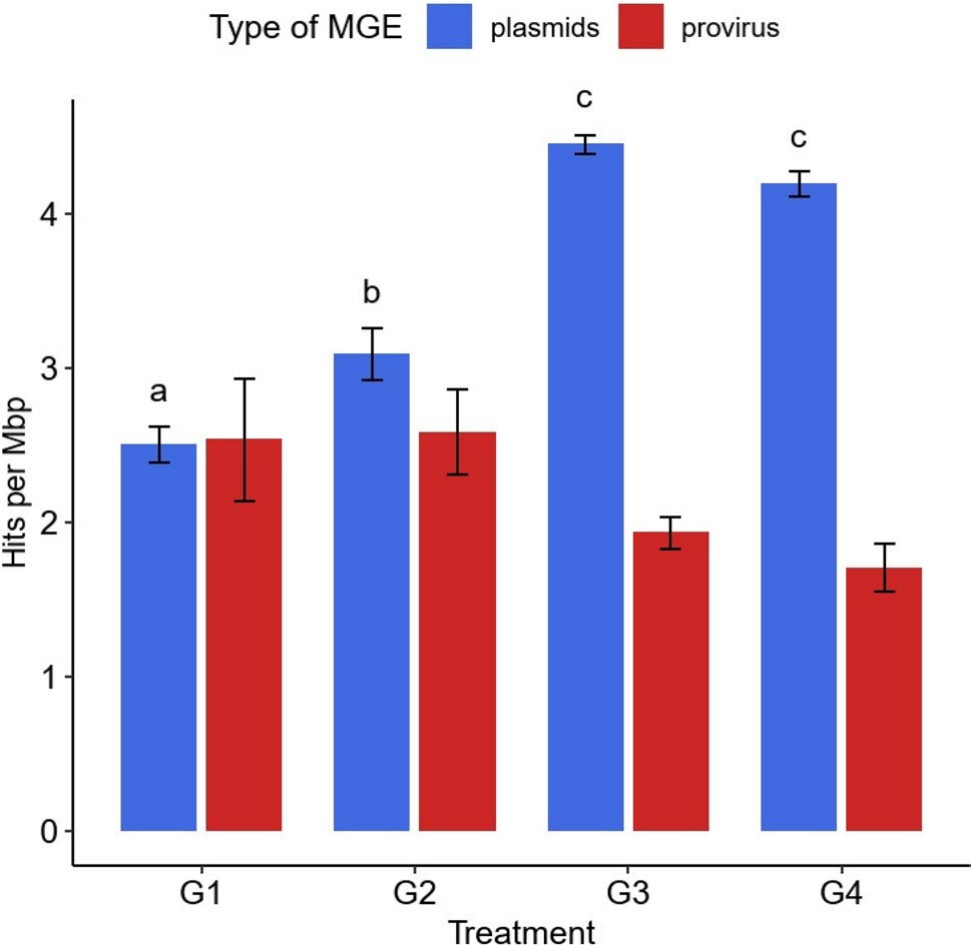
Normalized abundance of mobile genetic elements (MGEs) as plasmids and proviruses across green roof treatments. Abundance was normalized to the total number of base pairs to account for varying sequencing depth. Statistical significance was assessed using one-way ANOVA followed by Tukey’s HSD post-hoc. Different letters above bars indicate significant differences in plasmid abundance between treatments

Although ARGs were rarely plasmid-associated, the abundance of plasmids differed significantly across treatments (ANOVA; *P* = 6.06 × 10^− 6^; η^2^ = 0.96) (Fig. 4). In particular, samples from G3 and G4 showed a significantly higher abundance, clustering together into a distinct group (Tukey’s HSD; group C) compared to the samples of G1 (group a; 95% CI: [1.42, 2.46] & [1.17, 2.20]; *P* = 1 × 10^− 5^ and *P* = 1 × 10^− 5^ for G3 and G4, respectively) and G2 (group b; 95% CI: [0.84, 1.87] & [0.58, 1.62]; *P* = 1 × 10^− 4^ and *P* = 6 × 10^− 4^ for G3 and G4, respectively), with lower abundance. Conversely, the abundance of proviruses did not differ significantly across treatments (ANOVA; P = 0.101; η^2^ = 0.1 ) (Fig. 4).

Correlation analyses between abiotic parameters and ARG abundance (Fig. S4) showed that pH was negatively correlated with several ARGs, including *bacA*, *bla*, *helR*, *sugE* and *arr*. Microbial biomass carbon (C_mic_) and dissolved organic carbon (DOC) were positively correlated with multiple ARGs, particularly *bacA*, *bla*, *helR* and *arr*. Microbial nitrogen (N_mic_) was strongly negatively correlated with *tlmA* and *aph(3’)-II* and positively correlated with *bacA* and *arr*.

## Discussion

Although previous studies have assessed the urban resistome of various urban interfaces [28–30], little is known on the resistome of urban green infrastructure, such as green roofs, and the effects that management practices can have on it [19, 31]. In this study, we characterized the ARGs composition of urban green roof substrates under different management regimes, using long-read metagenomic sequencing, which enabled species-resolved profiling of ARGs and their association with plasmids and other mobile genetic elements [19], allowing us to distinguish between intrinsic resistances and traits with higher dissemination potential.

Across all treatments, we detected a relatively consistent set of 62 ARGs. These were dominated by resistance to naturally occurring antibiotics, such as bacitracin and rifamycin, which function via target modification and mixed mechanism (both target modification and efflux-type), respectively, and therefore, would not be expected to be targets of co-selection by pollutants, like heavy metals. Resistance to these compounds is widely recognized as intrinsic in soil microorganisms and reflects long-standing microbial competition rather than recent anthropogenic antibiotic exposure [2, 3]. Their prevalence across all treatments, including non-amended roofs, indicates that the green roof resistome is primarily shaped by environmental selection pressure.

Given the proximity to heavy traffic, it could have been expected that ARGs which utilize modified transporters or efflux systems would be co-selected with heavy metals, as observed in urban soils [6]. However, heavy metal concentrations remained below the thresholds established by supranational organizations (Table S4). However, this does not exclude potential microbial selection pressure, as such thresholds do not necessarily represent microbiological no-effect concentrations. Since we did not perform correlation analyses between heavy metal concentrations and ARG abundance, nor assess metal bioavailability or ARG–metal-resistance gene co-localization, heavy-metal-mediated co-selection cannot be ruled out. Consequently, multidrug efflux-type ARGs were not markedly abundant. Interestingly, most ARG observations belonging to the multidrug class were found in G4 samples, which might suggest that either shrubs [31] or intensive substrate addition played a role in this.

ARG composition differed significantly among management regimes, suggesting that the green roof resistome maybe sensitive to differences in design and maintenance. However, these patterns should be interpreted with caution, as the treatment areas also differed in rooftop location and establishment time, which may have influenced microclimated conditions, substrate development, vegetation establishment and microbial succession. The management type had a significant effect on ARG richness but not on normalized ARG abundance. In particular, the plots with green waste addition displayed a higher ARG richness. Similar patterns have been observed in soils amended with organic waste, where compost inputs restructured resistome composition without necessarily increasing ARG loads [14, 32]. The concomitant increase in bacterial biomass and diversity observed in amended treatments supports the interpretation that ARG enrichment reflects a larger and more metabolically active microbial community rather than enhanced resistance selection. Nevertheless, the dominance of bacitracin and rifamycin resistance genes contrasts with urban ground soils, where resistance to β-lactams, quinolones, and tetracyclines often predominates [33, 34]. This divergence likely reflects the distinct ecological constraints of green roofs, including nutrient limitation, desiccation stress, and intense microbial competition, which favour antimicrobial production and intrinsic resistance mechanisms [7, 11]. However, given the confounded experimental design, the observed ARG patterns should be interpreted as associations with distinct green roof settings rather than as direct effects of individual management practices.

Correlations between ARG abundance and soil properties further link resistome patterns to measured environmental drivers. Contrary to many reports showing positive relationships between pH and ARG abundance in agricultural and riparian soils [15], pH in this study was negatively correlated with several dominant ARGs. This suggests that in green roof substrates, pH effects may be secondary to substrate composition and organic matter quality. In contrast, microbial biomass carbon and dissolved organic carbon were positively correlated with multiple ARGs, consistent with previous findings that ARG abundance often correlates with microbial biomass [35]. Dissolved organic nitrogen showed strong positive correlations with bacitracin, β-lactam, and biocide resistance genes, aligning with evidence that nitrogen availability can indirectly influence resistome structure by stimulating microbial growth and competition [16]. Therefore, nutrient availability or stoichiometry might be important management tools to control ARG proliferation in urban sites.

Taxonomic assignment revealed that ARGs, and in particular enriched ARGs, were predominantly carried by non-pathogenic, environmentally associated genera such as *Nocardioides*, *Bradyrhizobium*, *Allosphingosinicella*, and *Sphingomicrobium*. These taxa are characteristic of compost, urban, and oligotrophic soils and are known to harbour intrinsic resistance traits and high metabolic versatility [36, 37]. Several of these genera are also capable of degrading complex organic pollutants, which may promote co-selection between resistance and detoxification pathways [6]. Remarkably, no ARGs were associated with ESKAPE pathogens, substantially reducing immediate public health concerns [4].

Horizontal gene transfer represents a key mechanism for ARG dissemination in urban environments [38, 39]. Although only two ARGs were detected on plasmids, the significant increase in plasmids abundance with green waste addition suggests mobilization potential. Similar decoupling between ARG abundance and mobility has been reported in amended soils, where increased gene transfer capacity may precede detectable ARG spread [33, 40]. While current risks appear low, a full mobility analysis, including the integration of transposons, integrons and ARG-MGE co-localisation would be required to fully evaluate dissemination potential.

## Conclusions

Overall, our findings demonstrate that the resistome of the studied urban green roof was primarily shaped by intrinsic environmental selection rather than anthropogenic pollution, whose ARGs were not carried by pathogenic bacteria, but rather environmentally associated ones. Moreover, the management played a key role in shaping the resistome. While green waste addition increased ARGs richness and the abundance of plasmids, the absence of ESKAPE pathogens and the prevalence of ARGs with target-modification and mixed mechanisms suggest low immediate public health risks. Our findings highlight the relevance of the soil resistome in this specific green roof, however, further comprehensive, multi-site studies are required before resistome-based assessments can be reliably integrated into urban green infrastructure planning and One Health risk management strategies. Moreover, although the MinION sequencing effectively identified the dominant ARGs and bacterial hosts, greater sequencing depth, such as with PromethION, could further refine the detection of rare ARGs or low-abundance pathogens. Likewise, the addition of more replicates per plot and more sites could strengthen the significance of our findings. 

## Supplementary Information

Supplementary Material 1.

## Data Availability

The datasets generated during the current study are available in the NCBI’s Sequence Read Archive under the BioProject ID PRJNA1424695. The underlying code for this study is available in GitHub and can be accessed via this link [https://github.com/rubmc97/green_roof_args].
