# Supplementary material for: Urban green roofs host intrinsic resistomes shaped by management but not dominated by pathogenic resistance
Source: BMC Microbiol. 2026 Aug 6;26:701. doi: 10.1186/s12866-026-05495-6 (PMC13445745; doi:10.1186/s12866-026-05495-6)
Supplement: Supplementary file 1 [file 12866_2026_5495_MOESM1_ESM.docx]

**Urban green roofs host intrinsic resistomes shaped by management but not dominated by pathogenic resistance**

Rubén Martínez-Cuesta^12*^; Alexandra Craighero^23^; Susanne Walch^2^; Brigitte Helmreich^4^; Michael Schloter^12^; Stefanie Schulz^2^

^1^Chair of Environmental Microbiology, TUM School of Life Sciences, Technical University of Munich, Emil-Ramann-Straße 2, 85354 Freising, Germany

^2^Research Unit Comparative Microbiome Analysis, Helmholtz Zentrum Munich, Ingolstädter Landstraße 1, 85764 Neuherberg, Germany

^3^Chair of Terrestrial Ecology, TUM School of Life Sciences, Technical University of Munich, Hans-Carl-von-Carlowitz-Platz 2, 85354, Freising, Germany

^4^Chair of Urban Water Systems Engineering, School of Engineering and Design, Technical University of Munich, Am Coulombwall 3, 85748 Garching, Germany

**Supplementary methods**

**Plant species composition**

The 5 bulb species that were sown in the plot under G2 treatment were: *Verbascum phoeniceum*, *Silene ameria*, *Silene galica*, *Silene nutans*, *Thymus praecox*.

The 12 bulb species that were sown in the plot under G3 treatment were: *Allium millenium*, *Allium tanguticum*, *Muscari neglectum*, *Muscari azureum*, *Allium carinatum*, *Tulipa batalini*, *Allium amplectens*, *Allium flavum*, *Allium oreophilum*, *Allium senescens*, *Muscari neglectum* and *Tulipa batalini*.

7 shrubs were planted in the plots under the G4 treatment. Those belonged to the three initial species that colonized the rooftop before the study: *Amelanchier ovalis*, *Genista tinctoria* and *Primula veris*.

**Physicochemical parameters and heavy metal concentrations**

Soil water content was determined gravimetrically by weighing 2 g of each sample before and after drying at 65 °C for 48 h. For pH determination, 5 g of soil were weighed and 25 mL of 0.01 M CaCl_2_ solution were added. The mixture was incubated for 24 h at 4°C before measuring with a pH meter (WTW Xylem Analytics Germany GmbH, Germany). For DOC, DON, C_mic_ and N_mic_ measurements, two subsamples of 5 g each were weighed into glass vials. One set was placed in a desiccator and fumigated with ethanol-free chloroform for 24 h. The second set of samples was extracted with 20 mL of 0.01 M CaCl_2_ by shaking during 40 min. The suspension was filtered through Whatman 595 ½ filter paper (Whatman, Germany) and stored at -20 °C. After fumigation, the chloroform-treated samples were extracted (20 mL 0.01 M CaCl_2_), filtered and stored in the same way. DOC and DON concentrations were measured with a DIMA-TOC 100 analyser (Dimatec Analysentechnik GmbH, Germany) in the unfumigated samples. Microbial C and N (C_mic_, N_mic_) were calculated by subtracting the measured DOC, DON values from the fumigated samples.

Total concentrations of lead (Pb), copper (Cu), and zinc (Zn) were determined from 3 g of mixed sample from each replicate. Samples were dried at 60°C for 48 h. Prior to analysis, samples underwent aqua regia digestion in accordance with EN 13346:2001 [1] protocols. Metal quantification was performed via Inductively Coupled Plasma Optical Emission Spectrometry (ICP-OES) following ISO 11885:2007 [2] standards.

**Bioinformatic analyses**

Barrnap v0.9 (https://github.com/tseemann/barrnap) was used to predict ribosomal RNA genes from the reads. Predicted 16S rRNA gene sequences were extracted and sequences flagged as partial were discarded. Taxonomic assignment and abundance estimation of the extracted 16S rRNA gene sequences were performed using emu [3] v3.5.3 and its reference database.

Estimated counts from the taxonomically classified 16S rRNA gene dataset were normalized using Total Sum Scaling (TSS) to account for differences in sequencing depth between samples. Differences in bacterial richness, defined as the number of observed species, between treatments were assessed using a one-way ANOVA test.

The β-diversity variation within samples from the same plot was assessed using the ‘betadisper’ function of the vegan package v2.6-4 [4]. Differences in distance to centroid were assessed with a one-way ANOVA test with the “anova_test” function of the rstatix v0.7.2 package, with prior residuals testing with the Levene’s test using the “leveneTest” function of the car v3.1-3 package and the Shapiro-Wilk test.

**Supplementary figures**

**Figure S1.** Pictures from the green roof plots sampled in September 2023. G1-G3 were constructed in 2018, G4 in 2020.

**Figure S3.** Beta dispersion (distance to centroid) across treatment groups. Boxplot representing the multivariate spread of ARGs composition based on Bray-Curtis distances.

**Figure S2.** Bacterial biomass and richness across plots in the green roof. **A** Bacterial biomass across treatments, estimated via microbial biomass carbon (C_mic_). **B** Bacterial richness across treatments, estimated as the number of observed species based on the 16S rRNA gene sequences classified with emu. Differences between treatments were assessed using one-way ANOVA tests, and with the Tukey’s HSD post-hoc test for pairwise comparisons in **A** after the significant ANOVA.

**Figure S4.** Correlation, estimated as Spearman’s ρ, between the abundance of the top 10 ARGs and the measured physicochemical parameters.

**Supplementary tables**

**Table S1.** Mean and standard deviation (sd) of the biogeochemical parameters measured at the different plots of the green roof: pH, moisture, dissolved organic carbon (DOC), microbial biomass carbon (C_mic_), dissolved organic nitrogen (DON) and microbial biomass nitrogen (N_mic_).

| **Treatment** | **pH** | **Moisture** | **DOC (µg/g)** | **C_mic_ (µg/g)** | **DON (µg/g)** | **N_mic_ (µg/g)** |
| --- | --- | --- | --- | --- | --- | --- |
| G1 | 7.23 ±0.09 | 18.1±9.97 | 79.4±7.49 | 24.2±18.17 | 7.58±0.71 | 2.44±1.35 |
| G2 | 7.43 ±0.03 | 11.2±4.34 | 71.1±5.35 | 136.3±100 | 9.22±1.01 | 35.9±16.7 |
| G3 | 7.21 ±0.11 | 17.5±5.88 | 109±37.7 | 555±242 | 6.95±1.37 | 6.82±3.16 |
| G4 | 7.14 ±0.07 | 17.5±7.52 | 97.1±8.88 | 350±140 | 14.4±7.97 | 29.0±5.27 |

**Table S2.** Summary of raw and processed long-read sequencing data for all samples. Metrics include the number of sequences, total length, average read length, and maximum read length, all in base pairs (bp), as well as average read quality as Phred Score and GC content.

| Reads | Treatment | Sequences number | Total length | Average length | Max length | Average quality | GC (%) |
| --- | --- | --- | --- | --- | --- | --- | --- |
| SQK-NBD114-96_barcode48.fastq | G1 | 295,593 | 634,765,661 | 2,147 | 60,553 | 14.64 | 62.5 |
| SQK-NBD114-96_barcode49.fastq | G1 | 316,560 | 598,407,608 | 1,890 | 63,507 | 14.41 | 64.22 |
| SQK-NBD114-96_barcode50.fastq | G1 | 551,781 | 1,001,003,406 | 1,814 | 31,805 | 14.52 | 63.45 |
| SQK-NBD114-96_barcode51.fastq | G2 | 553,385 | 1,123,987,326 | 2,031 | 26,720 | 14.55 | 63.42 |
| SQK-NBD114-96_barcode52.fastq | G2 | 561,766 | 1,304,194,023 | 2,322 | 182,364 | 14.66 | 62.34 |
| SQK-NBD114-96_barcode53.fastq | G2 | 761,618 | 1,814,052,404 | 2,382 | 313,368 | 14.57 | 64.91 |
| SQK-NBD114-96_barcode54.fastq | G3 | 759,200 | 2,487,825,487 | 3,277 | 62,158 | 14.88 | 64.17 |
| SQK-NBD114-96_barcode55.fastq | G3 | 819,190 | 2,610,749,945 | 3,187 | 59,475 | 14.86 | 63.5 |
| SQK-NBD114-96_barcode56.fastq | G3 | 720,194 | 2,335,430,138 | 3,243 | 37,201 | 14.92 | 64.45 |
| SQK-NBD114-96_barcode57.fastq | G4 | 451,150 | 1,273,304,435 | 2,822 | 41,357 | 14.84 | 63.06 |
| SQK-NBD114-96_barcode58.fastq | G5 | 951,751 | 3,093,788,713 | 3,251 | 84,368 | 14.86 | 63.95 |
| SQK-NBD114-96_barcode59.fastq | G6 | 644,649 | 2,148,927,925 | 3,334 | 63,233 | 14.93 | 63.19 |

**Table S3.** Detected antibiotic resistance gene (ARG) subtypes, their resistance mechanism (type), genomic location, and clinical relevance classification.

| **Type** | **Clinical relevance** | **Subtype** | **Carrier** |
| --- | --- | --- | --- |
| aminoglycoside | Clinical | *aac(2')-I* | chromosome |
| aminoglycoside | Clinical | *aac(3)* | chromosome |
| aminoglycoside | Clinical | *aac(3)-I* | chromosome |
| aminoglycoside | Clinical | *aac(3)-IV* | chromosome |
| aminoglycoside | Clinical | *aac(6')* | chromosome |
| aminoglycoside | Clinical | *ant(3'')-I* | chromosome |
| aminoglycoside | Clinical | *ant(6)* | chromosome |
| aminoglycoside | Clinical | *ant(9)* | chromosome |
| aminoglycoside | Clinical | *aph(3')* | chromosome |
| aminoglycoside | Clinical | *aph(3'')* | chromosome |
| aminoglycoside | Clinical | *aph(3')-II* | chromosome |
| rifamycin | Clinical | *arr* | chromosome |
| bacitracin | Non-clinical | *bacA* | chromosome |
| bacitracin | Non-clinical | *bceA* | chromosome |
| multidrug | Biocide/Multidrug | *bepE* | chromosome |
| beta-lactam | Clinical | *bla* | chromosome |
| beta-lactam | Clinical | *bla* | plasmid |
| beta-lactam | Clinical | *blaGRD33* | chromosome |
| beta-lactam | Clinical | *blaLRA* | chromosome |
| phenicol | Clinical | *catB* | chromosome |
| phenicol | Clinical | *cfr* | chromosome |
| phenicol | Clinical | *cml* | chromosome |
| capuramycin | Other | *cpr17* | chromosome |
| phenicol | Clinical | *cpt* | chromosome |
| trimethoprim | Clinical | *dfrB* | chromosome |
| trimethoprim | Clinical | *dfrB* | plasmid |
| defensin | Other | *dltA* | chromosome |
| defensin | Other | *dltC* | chromosome |
| multidrug | Biocide/Multidrug | *efpA* | chromosome |
| aminoglycoside | Clinical | *eis* | chromosome |
| macrolide-lincosamide-streptogramin | Clinical | *erm* | chromosome |
| fosfomycin | Clinical | *fos* | chromosome |
| rifamycin | Clinical | *helR* | chromosome |
| multidrug | Biocide/Multidrug | *lieA* | chromosome |
| colistin | Other | *lpxE* | chromosome |
| defensin | Other | *lysX* | chromosome |
| macrolide-lincosamide-streptogramin | Clinical | *macA* | chromosome |
| macrolide-lincosamide-streptogramin | Clinical | *macB* | chromosome |
| multidrug | Biocide/Multidrug | *mdtA* | chromosome |
| multidrug | Biocide/Multidrug | *mdtC* | chromosome |
| multidrug | Biocide/Multidrug | *mdtI* | chromosome |
| macrolide-lincosamide-streptogramin | Clinical | *mgt* | chromosome |
| multidrug | Biocide/Multidrug | *mmp* | chromosome |
| defensin | Other | *mprF* | chromosome |
| multidrug | Biocide/Multidrug | *norA* | chromosome |
| aminocoumarin | Non-clinical | *novA* | chromosome |
| macrolide-lincosamide-streptogramin | Clinical | *ole(C)-1* | chromosome |
| multidrug | Biocide/Multidrug | *oqxB* | chromosome |
| tetracycline | Clinical | *otr(A)* | chromosome |
| macrolide-lincosamide-streptogramin | Other | *pep* | chromosome |
| rifamycin | Clinical | *rox* | chromosome |
| rifamycin | Clinical | *rph* | chromosome |
| biocide | Biocide/Multidrug | *smr* | chromosome |
| biocide | Biocide/Multidrug | *sugE* | chromosome |
| multidrug | Biocide/Multidrug | *tap* | chromosome |
| tetracycline | Clinical | *tet* | chromosome |
| tetracycline | Clinical | *tet(V)* | chromosome |
| bleomycin | Non-clinical | *tlmA* | chromosome |
| colistin | Clinical | *ugd* | chromosome |
| glycopeptide | Clinical | *vanA* | chromosome |
| glycopeptide | Clinical | *vanH* | chromosome |
| glycopeptide | Clinical | *vanK* | chromosome |
| glycopeptide | Clinical | *vanX* | chromosome |
| macrolide-lincosamide-streptogramin | Clinical | *vat* | chromosome |

**Table S4.** Measurements of the heavy metals lead (Pb), copper (Cu), Zinc (Zn) from the different plots of the green roof and recommended values for soil according to the Food and Agriculture Organization of the United Nations (FAO) [5], the European Council (EC) [6] and the German Federal Soil Protection and Contaminated Sites Ordinance (BBodSchV) [7]. All results are expressed in mg/kg based on dry soil mass.

| **Treatment** | **Pb (mg/kg)** | **Cu (mg/kg)** | **Zn (mg/kg)** |
| --- | --- | --- | --- |
| G1 | 5.10 | 10.6 | 28.2 |
| G2 | 7.79 | 9.01 | 25.01 |
| G3 | 8.36 | 33.2 | 89.4 |
| G4 | 4.36 | 31.0 | 57.7 |
| FAO | 5-50 | 20-50 | 10-50 |
| EC | 40-70 | 50-100 | < 100 |
| BBodSchV (sand) | 40 | 20 | 60 |
| BBodSchV (silt & clay) | 70 | 40 | 150 |

**Supplementary references**

1. European Committee for Standardization. Characterization of Sludges—Determination of Trace Elements and Phosphorus—Aqua Regia Extraction Methods. Brussels; 2000.

2. Wells J, Kazakova O, Posth O, Steinhoff U, Petronis S, Bogart LK, et al. Standardisation of magnetic nanoparticles in liquid suspension. J Phys D Appl Phys. 2017;50:383003. https://doi.org/10.1088/1361-6463/aa7fa5.

3. Curry KD, Wang Q, Nute MG, Tyshaieva A, Reeves E, Soriano S, et al. Emu: species-level microbial community profiling of full-length 16S rRNA Oxford Nanopore sequencing data. Nat Methods. 2022;19:845–53. https://doi.org/10.1038/s41592-022-01520-4.

4. Oksanen J, Kindt R, Legendre P, O’Hara B, Simpson G, Solymos P, et al. The vegan package. 2008.

5. Food and Agriculture Organization of the United Nations. Maximum Limits for Dangerous Substances in Soil and Groundwater. 2004.

6. European Council. Council Directive 86/278/EEC on the protection of the environment, and in particular of the soil, when sewage sludge is used in agriculture. 1986.

7. Bundes-Bodenschutz- und Altlastenverordnung. Bundes-Bodenschutz- und Altlastenverordnung (BBodSchV). Germany; 2021.
